# Exploring the Impact of Gender and Age of Onset on Psoriasis Treatment Management

**DOI:** 10.3390/jcm14124090

**Published:** 2025-06-10

**Authors:** Tair Lax, Edia Stemmer, Noga Fallach, Guy Shrem, Michal Schreiber-Divon, Snait Ayalon, Eitan Giat, Inbal Mor, Mali Salmon-Divon

**Affiliations:** 1 Department of Molecular Biology, Ariel University, Ariel 4070000, Israel; 2 Fertility Clinic, North District, Clalit Health Services, Migdal HaEmek 2303001, Israel; 3 Special Education, Herzog Academic College of Education, Jerusalem 9426223, Israel; 4 Special Education, Bar-Ilan University, Ramat Gan 5290002, Israel; 5 Research Authority, Emek Medical Center, Afula 1834111, Israel; 6 The Department of Medicine, Sheba Medical Center, Ramat Gan 5265601, Israel; 7 Adelson School of Medicine, Ariel University, Ariel 4070000, Israel

**Keywords:** skin diseases, retrospective studies, sex factors, biological products

## Abstract

**Background**: Psoriasis is a chronic inflammatory skin disease characterized by a bimodal onset distribution, with cases categorized as early-onset or late-onset. While the prevalence of psoriasis is nearly equal between genders, men typically experience more severe forms of the disease, leading to differences in treatment approaches and clinical outcomes. The aim of this study was to investigate gender-based differences in treatment patterns among psoriasis patients, with a focus on how these differences vary by disease onset (early vs. late). **Methods**: A retrospective cohort study including individuals diagnosed with psoriasis between 1998 and 2022 through Clalit Health Services (CHS) in Israel. Gender-based differences in treatment patterns by psoriasis onset were analyzed using Chi-square and Fisher exact tests and survival analyses. **Results**: The disease onset showed a bimodal distribution among 3999 individuals, with women experiencing earlier onset compared to men (median age 37.2 vs. 40.1 years; *p* < 0.001). In early-onset psoriasis, men were significantly more likely than women to receive systemic (17.9% vs. 6.5%; *p* < 0.001) and biological therapies (3.8% vs. 1.6%; *p* = 0.005) and initiated these treatments earlier (*p* < 0.001). In contrast, no significant gender-based treatment differences were observed in late-onset cases. Regardless of gender, early-onset patients began phototherapy earlier than late-onset patients (*p* < 0.001). **Conclusions**: Our results suggest that disease onset timing may influence treatment decisions and highlight the need for a more personalized approach to psoriasis management that considers both gender and age of onset.

## 1. Introduction

Psoriasis is a chronic inflammatory skin disease that varies from mild to severe and affects both genders with similar prevalence, manifesting at any age [1]. However, the onset of psoriasis exhibits a bimodal age distribution [2], characterized by early onset before the age of 40 and late onset after the age of 40 [3]. Early-onset psoriasis is strongly associated with genetic factors, such as the HLA-Cw6 allele, a family history of the disease, and more severe and extensive cutaneous involvement [4,5,6,7]. Psoriasis treatment strategy is based on severity: mild cases typically involve topical therapies such as corticosteroids and vitamin D analogs, moderate to severe cases may require phototherapy or systemic treatments like methotrexate and cyclosporine, while severe cases often rely on biological therapies (biologics) targeting immune pathways like TNF-alpha and IL-17 inhibitors [8,9]. Regardless of severity, lifestyle modifications are essential for effective disease management [10]. Despite the similar prevalence between genders, men often experience more severe forms of psoriasis. Studies have demonstrated higher median Psoriasis Area Severity Index (PASI) scores in men compared to women [11], and men’s higher use of biological treatments reflects greater disease severity [12]. Additionally, a recent study from our group identified transcriptomic differences in psoriatic lesions between men and women, revealing sex-specific molecular pathways and immune cell variations that may contribute to the observed disparities in disease severity [13]. The reasons for these gender differences remain unclear but are thought to be multifactorial, involving hormonal, genetic, and psychosocial factors [14]. Our study utilized electronic health records (EHRs) from Clalit Health Services (CHS) in Israel to examine gender-based and onset-based differences in treatment patterns in order to improve personalized treatment approaches and patient care.

## 2. Materials and Methods

### 2.1. Data Source

The study is based on electronic health records extracted from the CHS database, the largest Health Maintenance Organization (HMO) in Israel, serving a population of more than 4.5 million people as of 2024. Data was extracted by the North District’s Research Data Center, using the Clalit Research Data sharing platform for de-identified data powered by MDClone (https://www.mdclone.com, accessed on 12 May 2022). The electronic health records were recorded between 1998–2022, (excluding some retroactive diagnoses from before 1998) and contained clinical and administrative data collected in hospitals (inpatient clinics and emergency room settings), primary care clinics, pharmacies, laboratories, and diagnostic and imaging centers. The data are also linked to national databases providing socio-demographic information related to patients and clinics. The data were specifically extracted from the CHS database focusing on inflammatory diseases.

### 2.2. Definition of Cohort

We defined the psoriasis cohort using a previously described algorithm [15] requiring each patient to have at least three records of psoriasis diagnosis codes. ICD-10 codes L40 and L44.8, and ICD-9 codes 696, 696.1, and 696.8 were defined as the optimal diagnosis codes for psoriasis. Patients with other immune-mediated inflammatory diseases (IMIDs), such as rheumatoid arthritis, ulcerative colitis, Crohn’s disease, and psoriatic arthritis, were excluded to minimize confounding factors. To ensure data accuracy, we excluded patients whose initial psoriasis diagnosis coincided with their first database entry, as this indicated retrospective data inclusion. Additionally, patients with any other psoriasis-related diagnoses before their first confirmed diagnosis were excluded to establish the true onset of psoriasis (Supplementary Table S1). A graphical representation of this psoriasis cohort selection is provided in Supplementary Figure S1.

### 2.3. Baseline Characteristics

We identified the onset date of psoriasis by the first recorded ICD-10 diagnosis code of L40 or L44.8 in the database. Patients were classified as either early-onset (diagnosed before age 40) or late-onset (diagnosed at age 40 or older). We assessed the proportion of individuals reporting smoking or physical activity, as well as BMI and obesity rates, using the measurements closest to the defined onset date. The obesity rate was calculated using BMI, with obesity defined as a BMI greater than 30. We evaluated the socio-economic distribution across three scale parameters: low, median, and high.

### 2.4. Treatments

The systemic medications included in the analysis were Acitretin (Neotigason), Methotrexate, Cyclosporine, and Otezela. The biological medications analyzed were Remicade, Enbrel, Humira, Stelara, Cosentyx, Taltz, Tremfya, Ilumya, and Skyrizi. No records for Bimzelx or Siliq were available in the database. Cimzia was administrated to only 2 patients in the psoriasis cohort and was excluded from the analysis.

### 2.5. Statistical Analysis

We represented continuous variables (age at onset and BMI) by median and IQR and analyzed them using the Mann–Whitney–Wilcoxon test. Categorical variables, representing proportions within a selected population (smoking, obesity, physical activity, and socioeconomic distribution), were tested using Chi-square test. For cases with missing patient data, we specified in parentheses the number of patients included in the analysis.

We used Fisher’s exact test to analyze differences in the proportions of biological, systemic, or phototherapy treatments between males and females by psoriasis onset. We calculated the proportion of patients with at least one record of the specified medication or phototherapy relative to the total number of patients in each gender category.

The time from psoriasis onset to initiation of systemic or phototherapy treatment was estimated using Kaplan–Meier survival curves to account for the varying follow-up times, with patients censored if they did not receive treatment during the observation period. (time was calculated from the date of the initial psoriasis diagnosis to the date of the last recorded entry in the database, indicating the end of record tracking for the patient). Patients who commenced treatment before their first diagnosis of psoriasis (43 out of 615 patients who received systemic treatment and 13 out of 240 patients who received phototherapy) and patients whose initial diagnosis occurred before 1 January 2000, were excluded from the survival analysis. Additionally, univariate and multivariate Cox proportional hazards regression models were applied to assess the association between demographic and clinical factors and the time to initiation of systemic, biological, and phototherapy treatments. The survival and Cox regression analyses were performed using the “ggsurvfit” and “survival” R packages. All statistical analyses were performed using R (version 4.2.3). A *p*-value < 0.05 was considered statistically significant.

### 2.6. Use of Generative AI Tools

During the preparation of this manuscript, the authors used OpenAI’s ChatGPT-3 and ChatGPT-4 to assist with language editing, phrasing, and refining the clarity of ideas.

## 3. Results

### 3.1. Patient Characteristics

A flow chart of the cohort selection based on the inclusion criteria is shown in Supplementary Figure S1. A total of 3999 patients met the inclusion criteria, of whom, 2613 (65.3%) were male and 1386 (34.7%) were female, indicating a higher proportion of male patients. The age distribution at psoriasis onset showed a bimodal pattern in both genders, with women tending to have an earlier onset compared to men (Supplementary Figure S2). The median follow-up period in the psoriasis cohort was approximately 11 years for males and 12 years for females (Supplementary Figure S3).

We analyzed the baseline characteristics between men and women (Table 1), finding that men in the entire cohort and in the early onset subgroup were diagnosed at an older age (*p* < 0.001) and had higher rates of smoking and higher BMI (*p* < 0.001). In contrast, obesity rates, physical activity, and socioeconomic distribution were similar between genders. Among late-onset patients, males had a significantly higher smoking rate, while all other parameters were similar between genders, except for a higher, non-statistically significant rate of obesity among women compared to men.

### 3.2. Comparison of Psoriasis Treatment Management and Time to Treatment Initiation Between Male and Female

**Figure 1:**
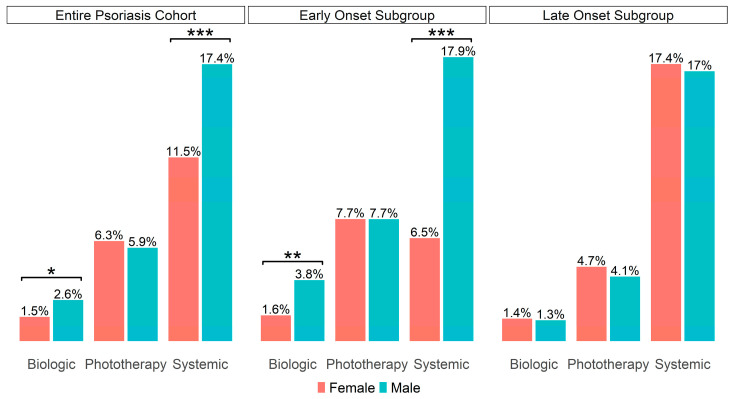
Gender-based comparison of psoriasis treatment. The percentages describe the proportion of patients who received at least one treatment out of the total number of patients for each gender. Analysis of the entire psoriasis cohort ((**left**), male: n = 2613, female: n = 1386) and the early-onset subgroup ((**middle**), male: n = 1305, female: n = 743), unveiled a distinct difference in rates of systemic and biological treatments. Conversely, examination of the late-onset subgroup ((**right**), male: n = 1308, female: n = 643), showed no significant gender-based difference. * *p* < 0.05, ** *p* < 0.01, *** *p* < 0.001. The absence of asterisks indicates no significant difference.

To evaluate potential gender-related differences in treatment patterns, and because objective measures of disease severity such as the (PASI) score were not available in our dataset, we compared the overall use of phototherapy, systemic, and biological treatments between men and women. In the absence of PASI scores, treatment type served as a proxy for severity, although it may not fully capture individual variations in disease burden. While no statistically significant difference was found in phototherapy rates between genders, males demonstrated higher biological and systemic treatment usage (*p* = 0.03, *p* < 0.001, respectively), as illustrated in Figure 1 (left). To understand the association between the age of disease onset and gender, we analyzed the early- and late-onset cohorts separately. In the early-onset subgroup, men exhibited significantly greater use of biological and systemic medications than women (*p* = 0.005, *p* < 0.001, respectively), with phototherapy rates remaining equal (Figure 1, middle). Conversely, in the late-onset subgroup, treatment rates, including systemic, biological, and phototherapy, showed no gender disparity (Figure 1, right).

**Figure 2:**
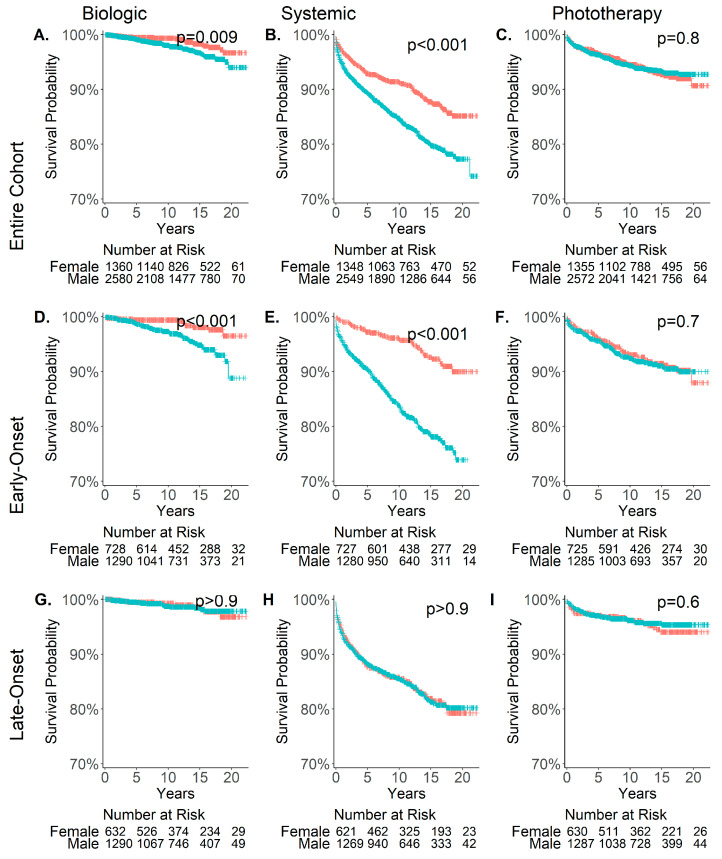
Time from the onset of psoriasis to the initiation of a new treatment among males and females. Time from psoriasis onset to the start of biological (left), systemic, (middle) and phototherapy (right) treatment in the entire psoriasis cohort (top—(**A**–**C**)), in the early-onset subgroup (middle—(**D**–**F**)) and late-onset subgroup (bottom—(**G**–**I**)) among male (blue) and female (red). Only patients who were diagnosed after 1 January 2000 and did not start treatment before their first psoriasis diagnosis were included in the analysis. Y-axis scale is (0.7, 1).

A similar trend was observed in a survival analysis (Figure 2) conducted to evaluate the time from the onset of psoriasis to the initiation of a new treatment (phototherapy, systemic, or biological). In the entire cohort, men received systemic and biological treatment significantly sooner than women, (Figure 2A,B) a pattern that was also evident in the early-onset subgroup (Figure 2D,E). No significant differences were observed in the late-onset subgroup (Figure 2G,H). This finding suggests that the significance observed in the entire cohort is primarily driven by the early-onset group. Regarding phototherapy, the time elapsed from the onset of psoriasis to the first phototherapy treatment showed no significant gender-based differences in either the early or late subgroup (Figure 2F,I).

Since biologics have been progressively integrated into clinical practice over the study period, we repeated the analysis, including only patients diagnosed after 2010, to assess the impact of increased biological availability on treatment patterns. In this analysis, early-onset men also received biological treatment significantly sooner than women (*p* = 0.03), while no significant differences were observed in the late-onset subgroup (Supplementary Figure S4).

### 3.3. Comparison of Time to Treatment Initiation Between Early-Onset and Late-Onset Patients

**Figure 3:**
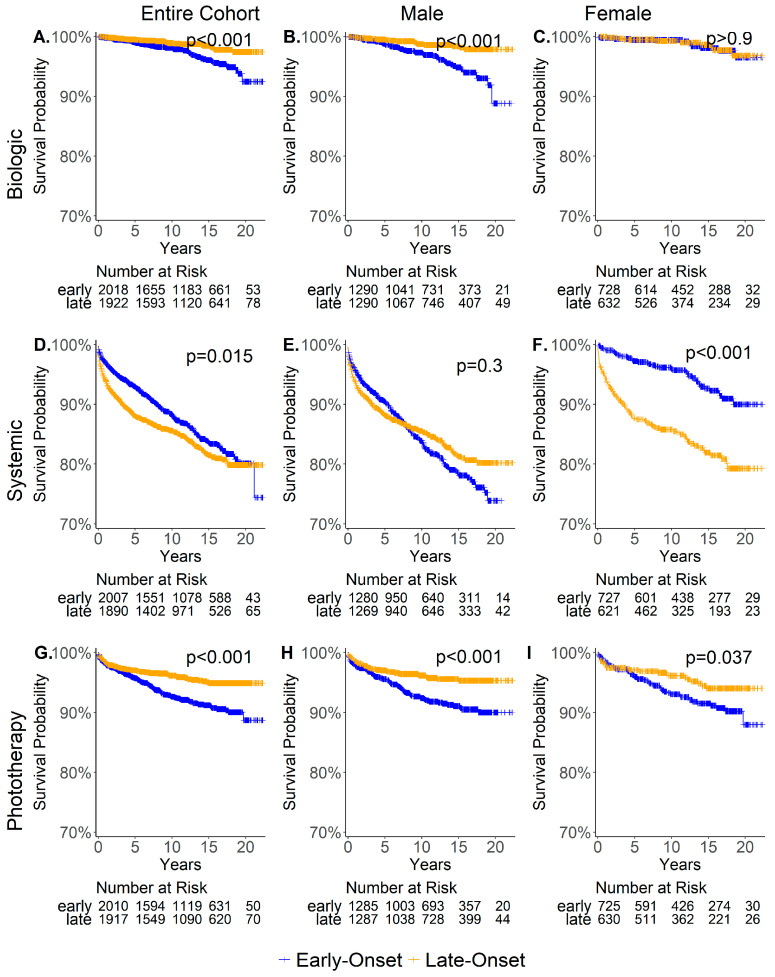
Time from the onset of psoriasis to the initiation of a new treatment among early and late-onset patients. Time from psoriasis onset to the start of biological (top—(**A**–**C**)), systemic, (middle—(**D**–**F**)) and phototherapy (bottom—(**G**–**I**)) treatment in the entire psoriasis cohort (left) male (middle) and female (right) subgroups, among early (blue) and late-onset (orange). Only patients who were diagnosed after 1 January 2000, and did not start treatment before their first psoriasis diagnosis were included in the analysis. Y-axis scale is (0.7, 1).

To further understand the different patterns of treatment initiation among early and late-onset patients, we compared the time from onset to treatment initiation between early-onset and late-onset males (Figure 3 middle), as well as between early-onset and late-onset females (Figure 3 right). Among males, early-onset patients received biological and phototherapy treatments significantly sooner than late-onset patients, although the time to systemic treatment initiation was similar between early and late-onset males (Figure 3B,E,H). For females, late-onset patients initiated systemic treatment earlier (Figure 3F), while there was no significant difference in the time to biological treatment initiation (Figure 3C). Similar to males, early-onset females received phototherapy sooner than late-onset females (Figure 3I).

### 3.4. Cox Regression Analysis

To further examine the influence of demographic and clinical variables on the timing of treatment initiation, we performed univariate and multivariate Cox proportional hazards regression analyses for biological, systemic, and phototherapy treatments. In the univariate analysis (Table 2), male gender was significantly associated with earlier initiation of both biological (HR = 1.9, 95% CI: 1.2–3.2, *p* = 0.01) and systemic treatments (HR = 1.7, 95% CI: 1.4–2.0, *p* < 0.0001), but showed no association with phototherapy initiation (*p* = 0.8). Late-onset psoriasis was associated with a reduced likelihood of initiating biological (HR = 0.4, 95% CI: 0.3–0.7, *p* = 0.0005) and phototherapy treatments (HR = 0.55, 95% CI: 0.4–0.7, *p* < 0.0001), but a slightly increased likelihood of initiating systemic therapy (HR = 1.23, 95% CI: 1.04–1.45, *p* = 0.02). Other significant predictors included smoking, higher BMI, obesity, and low socioeconomic status for systemic treatment, and physical activity, higher BMI, and medium socioeconomic status for phototherapy (Table 2).

In the multivariate analysis (Table 3), after adjusting for potential confounders, male gender remained a strong predictor of earlier initiation of biological (HR = 2.0, 95% CI: 1.2–3.3, *p* = 0.006) and systemic treatments (HR = 1.6, 95% CI: 1.3–1.9, *p* < 0.0001). Late-onset psoriasis continued to show a negative association with biological initiation (HR = 0.4, 95% CI: 0.3–0.7, *p* = 0.00035). Smoking (HR = 1.4, *p* = 0.0004) and low socioeconomic status (HR = 1.55, *p* = 0.002) were associated with earlier initiation of systemic therapy. In contrast, both higher BMI (HR = 0.96, 95% CI: 0.93–0.99, *p* = 0.046) and engagement in physical activity (HR = 0.48, 95% CI: 0.25–0.90, *p* = 0.03) were associated with a delayed initiation of phototherapy. These findings indicate that both gender and onset subgroups, along with lifestyle and socioeconomic factors, significantly influence treatment timelines in psoriasis.

## 4. Discussion

Over the past decade, studies evaluating the severity of affected areas in psoriasis have shown that men tend to have more extensive and severe manifestations of the disease than women [11,12,16,17]. Additionally, previous studies have highlighted distinct psychological [18], clinical [3,19], and genetic [5,20] characteristics between early-onset and late-onset psoriasis.

In line with other studies, we found that significantly more men received higher lines of therapy, suggesting a higher severity of psoriasis among men. However, after dividing the patients into early and late-onset subgroups, this trend was evident only in the early-onset subgroup. This is in agreement with previous studies indicating a notable difference in PASI scores between sexes in those under 30 [11,12]. This trend is further supported by our Cox regression analysis, which demonstrated that male gender was significantly associated with earlier initiation of both biological and systemic treatments, even after adjusting for potential confounders.

Our finding may stem from clinicians’ caution in prescribing systemic or biological treatments to women of childbearing age, as many immunosuppressive medications carry risks of teratogenic effects and potential complications during pregnancy and breastfeeding [21]. In the early-onset subgroup, 52.8% of women experienced one or more pregnancies after disease onset, which may explain the observed disparity in treatment. This may suggest that many women of childbearing age receive suboptimal treatment, highlighting the need for continued research on the safety of biologics during pregnancy and breastfeeding.

However, this explanation alone cannot account for the higher PASI scores or extent of involvement reported in men in other studies [11,12,16,17]. Therefore, given the median follow-up period for women of about 12 years (see Supplementary Figure S3), it is reasonable to assume that women with severe psoriasis would likely have been recommended treatment during intervals when pregnancy planning was not an issue. Additionally, certain biological drugs such as infliximab, adalimumab, and certolizumab pegol (Cimzia) may be considered for use during pregnancy and lactation in severe cases [22]. When comparing treatment patterns among women who experienced pregnancy after disease onset versus those who did not, we indeed observed a higher rate of systemic medication use among those who did not become pregnant. However, the rate of biological use was similar between the two groups (Supplementary Table S2). This suggests that biological differences, along with gender-based behaviors, could affect the course of the disease.

For example, variations in hormones and metabolism might influence disease severity compared to men. Both clinical and animal models have shown that hormones play a key role in mediating sex-specific differences in immune responses [23,24]. In psoriasis, these hormonal differences may influence the course of the disease. For example, the role of estrogen in human psoriasis is not fully understood, however, multiple studies suggest that estrogen has anti-inflammatory effects on the disease [25,26,27,28]. In addition, in a study of 47 pregnant psoriasis patients, 55% reported improvement during pregnancy, while postpartum, only 9% reported improvement [27]. Average monthly estrogen levels are significantly higher in younger women and decline with age [29]. Therefore, we hypothesized that estrogen plays a role in moderating psoriasis severity in young women, and the similarity in treatment rates observed between men and women in older age may be partly due to decreased estrogen levels in women. Supporting this, a study by Mowad et al. found that 48% of menopausal women experienced psoriasis exacerbation, while only 2% showed improvement [30]. Furthermore, later age at natural menopause and longer reproductive years were significantly associated with a reduced risk of late-onset psoriasis [31].

At the same time, it is important to highlight evidence suggesting that estrogen may also have pro-inflammatory effects on psoriasis, indicating a potential dual role for estrogen that varies depending on the context [32]. Additionally, women in the early-onset cohort had lower rates of smoking and lower BMI compared to both early-onset men and late-onset patients, which may also help explain the differences between genders. Apart from biological differences, behavioral aspects may also contribute to the differences between genders. Women are often more cautious about adopting new treatments, as they tend to perceive medical risks as higher [33] and have greater concerns about potential side effects, whereas men may be more open to innovative therapies.

While our primary focus was on treatment patterns, it is worth noting that clinical and demographic factors may also interact with age at disease onset in meaningful ways. Future research should explore these associations to better understand their impact on disease trajectory and treatment needs.

Our study has several limitations that should be acknowledged. Most notably, it lacked information on PASI scores—the gold standard for assessing disease severity and guiding treatment decisions. While treatment intensity was inferred based on therapy type, this cannot fully substitute for objective clinical severity data and limits our ability to correlate treatment disparities with actual disease burden. The data, recorded between 1999 and 2002, may also have gaps or incomplete records. Furthermore, the study included significantly more males than females, and the use of biologics was not consistently distributed across the years.

## 5. Conclusions

Our findings indicate that the timing of disease onset may play a role in treatment decisions, emphasizing the importance of a more personalized approach to psoriasis management that accounts for both gender and age at onset.

## Figures and Tables

**Table 1:** Comparison of baseline characteristics between males and females in the psoriasis cohort.

	Entire Psoriasis Cohort				Early-Onset Subgroup				Late-Onset Subgroup			
	Total (n = 3999)	Male (n = 2613)	Female (n = 1386)	*p*	Total (n = 2048)	Male (n = 1305)	Female (n = 743)	*p*	Total (n = 1951)	Male (n = 1308)	Female (n = 643)	*p*
Onset age, Median (IQR)	39.1 (26.6–54.4)	40.1 (28.1–54.5)	37.2 (24.0–54.1)	<0.001 ^b^	26.8 (19.8–32.5)	28.1 (21.6–33.1)	24.7 (17.5–31.3)	<0.001 ^b^	54.7 (47.8–62.2)	54.5 (47.8–62.1)	55.2 (48.1–62.3)	0.9 ^b^
Smoking % (n)	35.2 (3911)	40.9 (2558)	24.5 (1353)	<0.001 ^a^	27.5 (1996)	32.6 (1275)	18.3 (721)	<0.001 ^a^	43.3 (1915)	49 (1283)	31.6 (632)	<0.001 ^a^
Physical Activity % (n)	17.3 (2160)	18.4 (1321)	15.5 (839)	0.1 ^a^	13.4 (925)	14.6 (522)	11.9 (403)	0.3 ^a^	20.2 (1235)	20.9 (799)	18.8 (436)	0.4 ^a^
BMI, Median (IQR, n)	26 (22.6– 29.8, 3902)	26.3 (23.3–29.8, 2550)	25.3 (21.6–29.7, 1352)	<0.001 ^b^	23.9 (20.8–27.4, 1996)	24.4 (21.6–27.8, 1272)	22.7 (19.9–26.5, 724)	<0.001 ^b^	28 (25.1–31.4, 1906)	28.1 (25.3–31.1, 1278)	27.7 (24.4–32.5, 628)	0.8 ^b^
Obese (BMI > 30) % (n)	24.1 (3902)	24 (2550)	24.5 (1352)	0.7 ^a^	14.7 (1996)	15.3 (1272)	13.5 (724)	0.3 ^a^	34.1 (1906)	32.6 (1278)	37.1 (628)	0.055 ^a^
Socioeconomic Distribution				0.06 ^a^				0.051 ^a^				0.87 ^a^
Socioeconomic High % (n)	23.7 (3933)	24.4 (2574)	22.6 (1359)		22.9 (2016)	23.9 (1289)	21.0 (727)		24.7 (1917)	24.8 (1285)	24.4 (632)	
Socioeconomic Medium % (n)	61.5 (3933)	61.8 (2574)	60.9 (1359)		59.7 (2016)	60.1 (1289)	59.0 (727)		63.4 (1917)	63.5 (1285)	63.1 (632)	
Socioeconomic Low % (n)	14.7 (3933)	13.8 (2574)	16.5 (1359)		17.4 (2016)	16 (1289)	19.9 (727)		11.9 (1917)	11.7 (1285)	12.5 (632)	
One or more pregnancies after onset %			28.8 (1386)				52.8 (743)				1.1 (643)	

^a.^ Chi-squared test. ^b.^ Mann–Whitney–Wilcoxon test. (n) represents the number of patients included in the analysis.

**Table 2:** Univariate Cox proportional hazard regression analysis for time to initiation of biological, systemic, and phototherapy treatments.

	Biological				Systemic				Phototherapy			
	n	Hazard Ratio (HR)	95% CI	*p*-Value	n	Hazard Ratio (HR)	95% CI	*p*-Value	n	Hazard Ratio (HR)	95% CI	*p*-Value
Late-Onset	3940	0.4	0.3–0.7	0.0005	3897	1.23	1.04–1.45	0.02	3927	0.55	0.4–0.7	<0.0001
Gender-Male	3940	1.9	1.2–3.2	0.01	3897	1.7	1.4–2	<0.0001	3927	0.97	0.74–1.3	0.8
Smoking	3855	1.1	0.7–1.7	0.7	3813	1.5	1.3–1.8	<0.0001	3845	1.1	0.84–1.45	0.5
Physical Activity	2121	0.7	0.3–1.7	0.4	2096	0.75	0.5–1	0.07	2115	0.46	0.24–0.88	0.02
BMI	3849	1.01	0.98–1.05	0.5	3808	1.02	1.01–1.04	0.0004	3838	0.96	0.94–0.98	0.001
Obese (BMI > 30)	3849	1.3	0.8–2.08	0.3	3808	1.3	1.07–1.5	0.008	3838	0.9	0.66–1.2	0.5
Socioeconomic Low	3875	1.7	0.86–3.4	0.1	3832	1.5	1.2–2	0.002	3862	0.7	0.45–1.06	0.09
Socioeconomic Medium	3875	1.2	0.7–2.1	0.45	2832	1.2	0.99–1.5	0.06	3862	0.65	0.5–0.9	0.003

(n) represents the number of patients included in the analysis.

**Table 3:** Multivariate Cox proportional hazards regression analysis for time to initiation of biological, systemic, and phototherapy treatments.

	Hazard Ratio (HR)	95% CI	*p*-Value
**Biological (n = 3940)**			
Late-Onset	0.4	0.3–0.7	0.00035
Gender-Male	2	1.2–3.3	0.006
**Systemic (n = 3722)**			
Late-Onset	1.05	0.9–1.25	0.6
Gender-Male	1.6	1.3–1.9	<0.0001
Smoking	1.4	1.15–1.6	0.0004
BMI	1	0.99–1.03	0.3
Obese (BMI > 30)	1.1	0.8–1.5	0.5
Socioeconomic Low	1.5	1.2–2	0.002
Socioeconomic Medium	1.2	0.96–1.5	0.1
**Phototherapy (n = 2075)**			
Late-Onset	0.7	0.5–1.04	0.07
BMI	0.96	0.93–0.99	0.046
Physical Activity	0.48	0.25–0.9	0.03
Socioeconomic Low	0.64	0.3–1.2	0.2
Socioeconomic Medium	0.83	0.5–1.3	0.4

## Data Availability

The data used in this study were accessed under a specific data-sharing agreement with Clalit Health Services (CHS), Israel. Access to these data is restricted to protect patient confidentiality and is available only to researchers who obtain permission from CHS following submission and approval of a detailed research protocol. Data analyses were conducted within the CHS research room as required by CHS policy. Researchers interested in accessing the data or computing code should contact Clalit Health Services directly to inquire about the data access procedure.

## Supplementary Materials

The following supporting information can be downloaded at: https://www.mdpi.com/article/10.3390/jcm14124090/s1, Figure S1: A graphical representation of the psoriasis cohort selection process; Table S1: Psoriasis-related diagnoses which not included in the psoriasis cohort criteria; Figure S2: Distribution of males and females by age at first diagnosis of psoriasis; Figure S3: Distribution of time from psoriasis onset to the patient’s last recorded date in the database; Figure S4: Time from Psoriasis Onset to Treatment Initiation Among Males and Females Diagnosed After 2010; Table S2: Treatment Patterns in Women With Versus Without Pregnancy Following Psoriasis Onset.

## Abbreviations

The following abbreviations are used in this manuscript:

CHS	Clalit Health Services
PASI	Psoriasis Area Severity Index
EHR	Electronic Health Records
HMO	Health Maintenance Organization
IMIDs	Immune-Mediated Inflammatory Diseases
