# Supplementary material for: Exploring the Impact of Gender and Age of Onset on Psoriasis Treatment Management
Source: J Clin Med. 2025 Jun 10;14(12):4090. doi: 10.3390/jcm14124090 (PMC12193760; doi:10.3390/jcm14124090)
Supplement: Supplementary file 1 [file jcm-14-04090-s001.zip › jcm-3652566-supplementary.pdf]

Supplementary Materials

Table S1. Psoriasis-related diagnoses which not included in the psoriasis cohort criteria. .

Diagnosis	ICD10	ICD9/other codes
Impetigo Herpetiformis	L40.1	6943
Parapsoriasis	L41	6962
Intertrigo Psoriasis	L53.8	6958
Poikikoderma Vasculare Atrophicans	unknown	6962
Psoriasis w/wo Arthropathy	unknown	S91

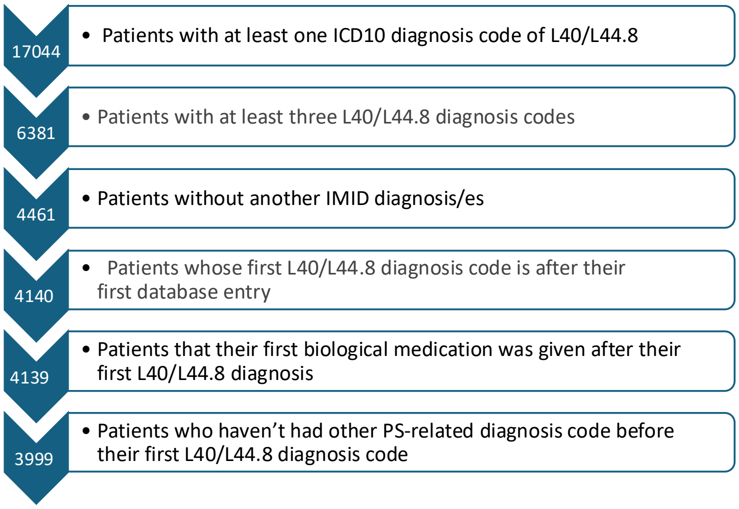

Figure S1. A graphical representation of the psoriasis cohort selection process

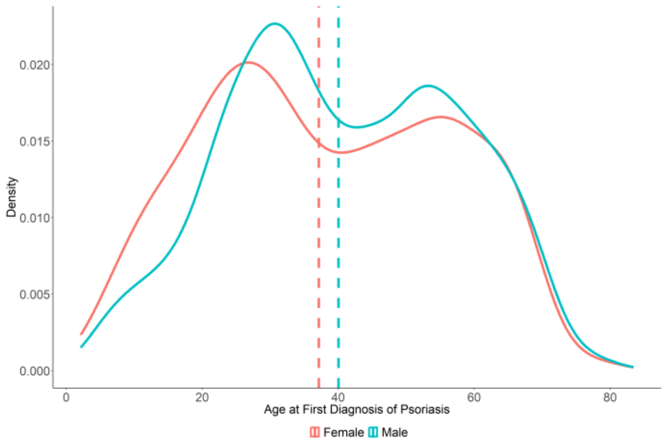

Figure S2. Distribution of males and females by age at first diagnosis of psoriasis. The dashed lines indicate the medians for males and females.

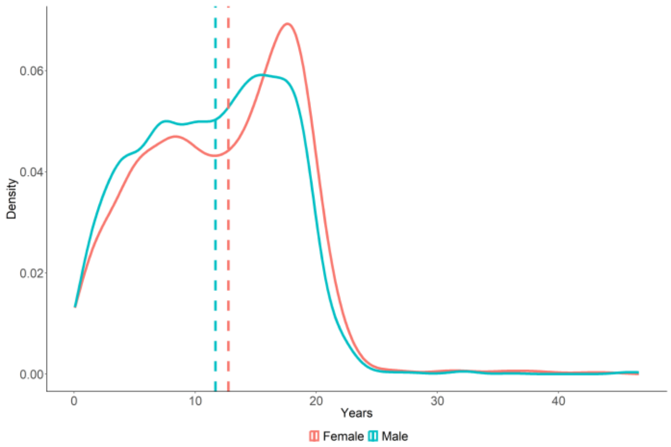

Figure S3. Distribution of time from psoriasis onset to the patient's last recorded date in the database. The dashed lines indicate the medians for males and females.

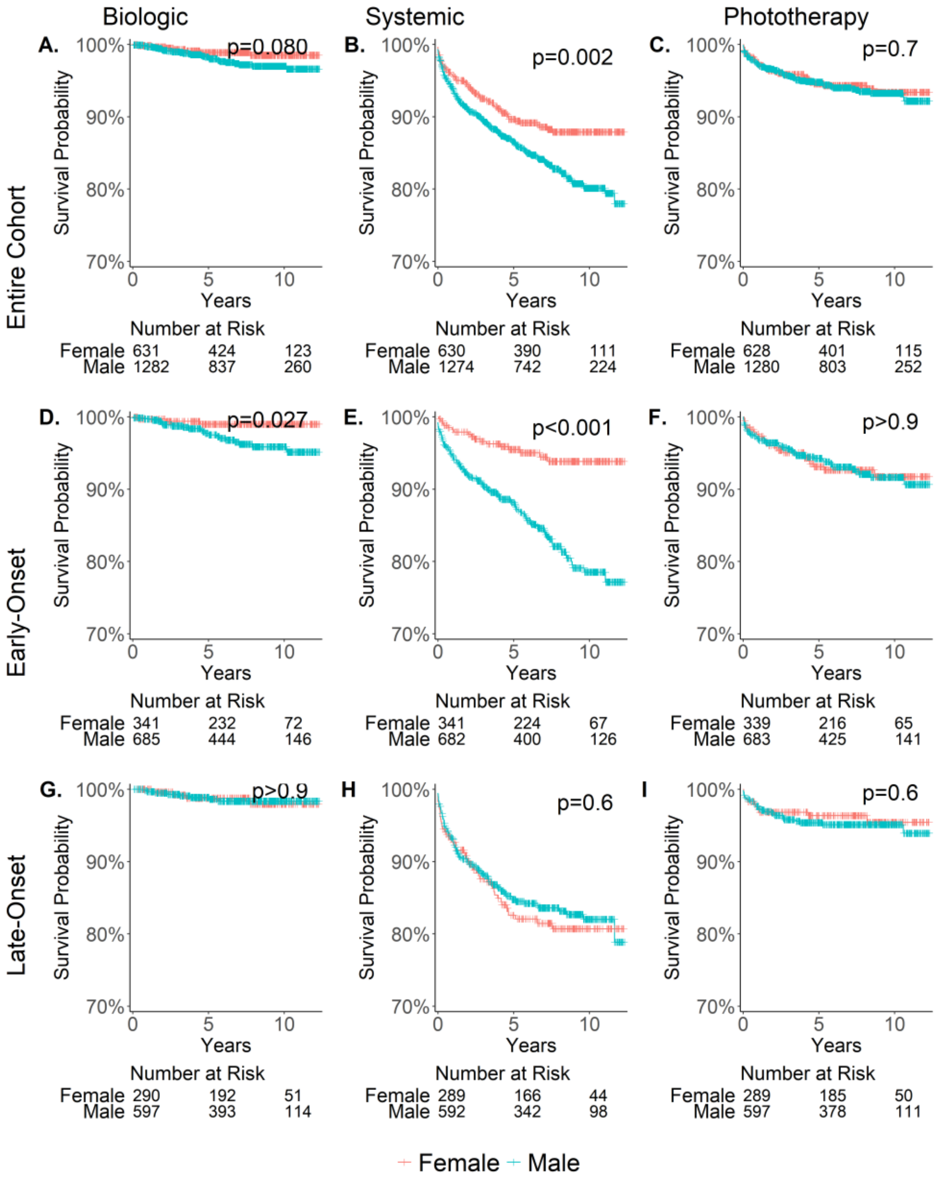

Figure S4. Time from Psoriasis Onset to Treatment Initiation Among Males and Females Diagnosed After 2010. Time from psoriasis onset to the start of biologic (Left), systemic, (Middle) and phototherapy (Right) treatment in the entire psoriasis cohort (Top - A, B, C), in the early-onset subgroup (Middle - D, E, F) and late-onset subgroup (Bottom - G, H, I) among male (blue) and female (red). Only patients who were diagnosed after January 1, 2010 and did not start treatment before their first psoriasis diagnosis were included in the analysis. Y axis scale is (0.7, 1)

Table S2. Treatment Patterns in Women With Versus Without Pregnancy Following Psoriasis Onset

	Women Who Experienced One or More Pregnancies After Psoriasis Onset (n=399)	Women Who Did Not Experienced Pregnancies After Psoriasis Onset (n=987)	P value
Biologic treatment* %	1.5	1.52	1[a]
Systemic treatment* %	7.02	12.87	0.002[a]
Phototherapy treatment* %	8.3	5.3	0.047[a]

a. Chi-squared test

* Treatments after disease onset
